# Depression and violence: a Swedish population study

**DOI:** 10.1016/S2215-0366(14)00128-X

**Published:** 2015-10

**Authors:** Seena Fazel, Achim Wolf, Zheng Chang, Henrik Larsson, Guy M Goodwin, Paul Lichtenstein

**Affiliations:** a Department of Psychiatry, University of Oxford, Oxford, UK

## Abstract

**Background:**

Depression increases the risk of a range of adverse outcomes including suicide, premature mortality, and self-harm, but associations with violent crime remain uncertain. We aimed to determine the risks of violent crime in patients with depression and to investigate the association between depressive symptoms and violent crime in a cohort of twins.

**Methods:**

We conducted two studies. The first was a total population study in Sweden of patients with outpatient diagnoses of depressive disorders (n=47 158) between 2001 and 2009 and no lifetime inpatient episodes. Patients were age and sex matched to general population controls (n=898 454) and risk of violent crime was calculated. Additionally, we compared the odds of violent crime in unaffected half-siblings (n=15 534) and full siblings (n=33 516) of patients with the general population controls. In sensitivity analyses, we examined the contribution of substance abuse, sociodemographic factors, and previous criminality. In the second study, we studied a general population sample of twins (n=23 020) with continuous measures of depressive symptoms for risk of violent crime.

**Findings:**

During a mean follow-up period of 3·2 years, 641 (3·7%) of the depressed men and 152 (0·5%) of the depressed women violently offended after diagnosis. After adjustment for sociodemographic confounders, the odds ratio of violent crime was 3·0 (95% CI 2·8–3·3) compared with the general population controls. The odds of violent crime in half-siblings (adjusted odds ratio 1·2 [95% CI 1·1–1·4]) and full siblings (1·5, 95% CI 1·3–1·6) were significantly increased, showing some familial confounding of the association between depression and violence. However, the odds increase remained significant in individuals with depression after adjustment for familial confounding, and in those without substance abuse comorbidity or a previous violent conviction (all p<0·0001). In the twin study, during the mean follow-up time of 5·4 years, 88 violent crimes were recorded. Depressive symptoms were associated with increased risk of violent crime and a sensitivity analysis identified little difference in risk estimate when all crimes (violent and non-violent) was the outcome.

**Interpretation:**

Risk of violent crime was increased in individuals with depression after adjustment for familial, sociodemographic and individual factors in two longitudinal studies. Clinical guidelines should consider recommending violence risk assessment in certain subgroups with depression.

**Funding:**

Wellcome Trust and the Swedish Research Council.

## Introduction

Depression is associated with increased risk of a wide range of adverse outcomes, including reduced life expectancy,^1^ suicide,^2^ self-harm,^3^ acute myocardial infarction,^4^ and a worse prognosis for comorbidities, such as heart disease and diabetes.^5^, ^6^ Clinical experience and expert opinion^7^ also suggest an association with the risk of perpetrating violence, including homicide in male perpetrators.^8^ Consistent with this, community surveys in the UK,^9^ register-based investigations in Australia,^10^ and cohort studies in the USA^11^ and New Zealand^12^ report a link with violent outcomes. However, this finding is not consistent and no association was identified in a recent US longitudinal study with lifetime^13^ or past year^14^ diagnoses. Moreover, in studies showing associations, they have been largely confounded by comorbid alcohol or drug use^13^ or sociodemographic factors,^15^ or primarily noted in individuals with psychotic depression.^16^

The probable reason for these inconsistencies could be that many influential studies have included large proportions of inpatients, where the actual reason for admission might have been risk of violence to others, suicidality, psychosis, or comorbid substance abuse. Because these are strong risk factors for violence,^17^ they will amplify and perhaps explain any effects. Some studies have tried to control for these confounders, but none to our knowledge have also adjusted for familial effects. Familial effects could be a further explanation for the reported association with depression and could arise from common genetic predisposition or shared early environmental adversity. Mediation of mechanisms such as impulsivity and mood instability could be important as common causes of both depression and violence.^18^

To clarify these uncertainties, we conducted two complementary studies that benefit from use of databases available for research in Sweden. In the first, we longitudinally followed up patients with an index diagnosis of depression to determine risks of violent crime; only outpatients were included to avoid the probable biases associated with inpatient samples. Risks of violent crime were also investigated in non-depressed siblings to determine the extent of familial confounding, and a comparison was made with risks from suicide mortality. In the second study, we investigated the association between depressive symptoms and violent crime in a cohort of twins. These studies accordingly control for the major confounds we identify in the existing literature. Because clinical guidelines are inconsistent about assessment and management of violence risk in major depression, and lack information about risk factors,^19^, ^20^ by contrast to self-harm and suicide for which risk assessment is clearly highlighted in guidelines^19^, ^20^, ^21^ and expert opinion,^22^ we investigated such rates in the same cohort to compare risks across outcomes where clinical guidelines provide differing recommendations.

## Methods

### Study design and participants

In this total population study, we linked several longitudinal, nationwide Swedish Patient Register: the National Patient, the Multi-Generation, the National Cause-of-Death, the Swedish Twin, and the National Crime Registers. The Multi-Generation Register connects every person born in Sweden in or after 1933 and ever registered as living in Sweden after 1960 to their parents.^23^ Similar information exists for those immigrants who became citizens of Sweden before age 18 years, together with one or both parents. Linkage of registers is possible because all residents including immigrants have a unique ten-digit personal identification number that is used in all national registers. We selected a cohort of individuals born between Jan 1, 1958, and Dec 31, 1994, who were followed from Jan 1, 2001, to the end of follow-up on Dec 31, 2009. National outpatient coverage in the Patient Register started on Jan 1, 2001, which was the reason that we started our follow-up at that time.

Using the Multi-Generation Register, we also identified patients with depression who had siblings and half-siblings without depression during the same period.

Using the Swedish Twin Register, we identified young adult to middle-aged (aged 18–47 years) twins born between Jan 1, 1959, and Dec 31, 1986, who had participated in the Study of Twin Adults: Genes and Environment (STAGE)^24^ or the Swedish Twin study of CHild and Adolescent Development (TCHAD).^25^ In total, 23 020 individuals from 15 298 twin pairs (5574 monozygotic and 9724 dizygotic pairs) were included in our study. We determined zygosity with DNA testing or validated zygosity questionnaires.

Cases with depression were identified from the National Patient Register as having at least two outpatient episodes between Jan 1, 2001, and Dec 31, 2009, according to International Classification of Diseases [ICD]-10 (codes F32–F33·9).^26^ We excluded individuals with inpatient episodes of depression to avoid reverse causality (because violence and aggression might precipitate admission), and those with inpatient or outpatient diagnoses of schizophrenia, schizophrenia-spectrum, and bipolar disorder between Jan 1, 1969, and Dec 31, 2009.

In the twin study, we measured depressive symptoms with a short form of the Center for Epidemiologic Studies Depression (sCESD) scale.^27^ The sCESD scale included 11 items, and every item was rated on a 4-point scale (0=not at all or almost not at all; 1=rather rarely; 2=quite often; 3=all the time or almost all the time). A sum score was created based on the 11 items, with good reliability (Cronbach's α=0·86). 21 451 (93·2%) of the twins answered the questionnaires in 2005 (22 in 2004 and 1547 in 2006), and they were all followed for any outcome through linkage to the Crime Register.

The Regional Ethics Committee at the Karolinska Institutet approved the study (2009/939-31/5). Data were merged and anonymised by an independent government agency (Statistics Sweden), and the code linking the personal identification numbers to the new case numbers was destroyed after merging, so informed consent was not required.

### Diagnostic validity

Data from the Swedish Patient Register diagnoses have good to excellent validity for a range of conditions, including bipolar disorder^28^ and schizophrenia.^29^ Overall, the positive predictive value of the inpatient register, in a recent review, was identified to be 85–95% for most diagnoses.^30^ For the purpose of this study, we examined the validity of diagnoses of depression in a separate sample of patients with depression, by comparing concordance rates between patient register diagnoses (as we have used) and another clinical register that provided standardised consensus diagnoses involving comprehensive court-ordered multidisciplinary evaluations during 4 weeks in inpatient settings^31^—these acted as a gold standard. In this sample of 3059 patients assessed between 1996 and 2001, we noted fair to moderate agreement (κ of 0·32; 88% full agreement).

The mean sCESD depression score of twins who had a lifetime diagnosis of depression from the National Patient Register (mean score 13·1, 95% CI 12·7–13·6) was substantially higher than that of twins without a diagnosis of depression (7·0, 6·9–7·1).

In terms of reliability, in a previous register-based study, only around 1% (13 669 of 1 421 765) had missing personal identification numbers.^32^

### Measures

Data for convictions for violent crime between Jan 1, 1972, and Dec 31, 2009, were retrieved for all individuals in the cohort from the National Crime Register, which includes conviction data for everyone aged 15 years (the age of criminal responsibility) and older. These data were extracted both before (as covariate) and after diagnosis (as outcome) of depression. Conviction of a violent offence was defined as homicide and attempted homicide, aggravated assault (an assault that is life-threatening or leads to severe bodily harm), common assault, robbery, arson, any sexual offence (rape, sexual coercion, child molestation, and sexual harassment [including indecent exposure]), and illegal threats or intimidation.^32^ Conviction data were used because the Criminal Code in Sweden determines that individuals are convicted as guilty regardless of mental illness (ie, being judged as not guilty by reason of insanity is not an option). Thus, conviction data included individuals who received custodial or noncustodial sentences and people transferred to forensic psychiatric hospital. Furthermore, conviction data included individuals cautioned or fined (which are more likely to be used in juvenile cases). Additionally, although certain factors might affect sentencing, plea-bargaining at the conviction stage is not part of the Swedish legal system.^33^ Therefore, conviction data more accurately reflect the extent of officially resolved criminality in the population. The Crime Register has total national coverage—only 0·05% of all registered convictions had incomplete personal identification numbers during the years 1988–2000.^32^

Data for cause of death were retrieved for all individuals who died between Jan 1, 1969, and Dec 31, 2009. The Cause of Death Register is based on death certificates and covers more than 99% of all deaths.^34^ Suicides included undetermined deaths (ICD codes Y10–Y34) because their exclusion would underestimate actual rates.^35^ Data for self-harm were retrieved from the National Patient Register, and included certain (ICD codes X60–X84) and uncertain self-harm (ICD codes Y10–Y34).

### Sociodemographic and psychiatric covariates

Family disposable income at age 15 years (divided into thirds) was used as a proxy for income, and used as a dichotomous variable (lowest tertile *v*s top two tertiles). If this was unavailable, family disposable income, at age 16 years was used or until the age when it became available. Single marital status was defined as being unmarried at first diagnosis. Immigrant status was defined as being born outside of Sweden. Imputation or other methods were not used to replace missing family income data (0·7%). No other data were missing.

Drug and alcohol use disorders were defined with inpatient (1969–2009) and outpatient (2001–2009) primary or secondary diagnoses of alcohol or drug abuse or dependence (ICD–8: 303, 304; ICD–9: 303, 304, 305·1, 305·9; ICD–10: F10–F19, except x·5). The diagnostic validity of this secondary diagnosis is moderate.^36^

### Statistical analysis

For every patient, up to 20 general population control individuals without any diagnosis of depression were matched individually by birth year and sex. We estimated the association with having been diagnosed with depression, as per related work,^36^, ^37^ using matched controls, with the clogit command in Stata (version 12.1; StataCorp). The clogit command fits conditional (fixed effects) logistic regression models to matched case-control groups. We included two confounders (low family income and immigrant status) on theoretical grounds, based on related work in severe mental illness,^36^, ^38^ and also tested whether they were each independently associated with either case or control and outcome measures, respectively, in univariate analyses at 5% level of significance.^39^ We also conducted stratified subanalyses by sex, previous criminality, self-harm history, and drug and alcohol abuse.

To account for possible familial confounding, we undertook additional analyses with unaffected full siblings and half-siblings of patients as controls. In these analyses, we identified full siblings (n=33 519), paternal half-siblings (n=8734), and maternal half-siblings (n=6800), and these individuals were each compared with 20 age-matched and sex-matched general population controls with matched conditional logistic regression. Analyses were adjusted for low family income and immigrant status, and odds ratios of violence were reported.

We then compared patient analyses to sibling analyses using ratios of odds ratios.^40^ The ratios of odds ratios takes into account the increased risk of violence in unaffected siblings (figure). A ratio of odds ratios of 1·0 would mean that the risk of violence in those with depression (compared with the general population) is the same as the risk in their unaffected siblings (compared with the general population)—ie, the association between depression and violence is fully confounded by environmental and genetic factors shared by siblings.

**Figure:**
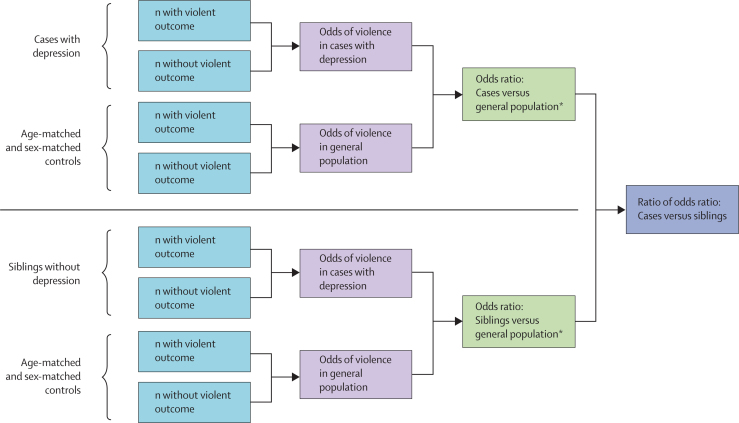
Ratio of odds ratio n=number. *Adjusted for low family income and born abroad.

We did four sensitivity analyses on the main sample. First, we examined those with at least one inpatient diagnosis between 2001 and 2009 (ie, patients who are excluded from the main analyses), and compared risk of violent crime with age-matched and sex-matched population controls. Adjustments were made for income and immigrant status.

Second, we excluded patients with depression with comorbid personality disorder. Comorbid personality disorder is common in patients with depression^41^ and is associated with violence,^42^ and hence excluding it would further test our primary hypothesis. Third, we restricted violent crime to specific outcomes for which interpersonal violence is known to have occurred—homicide, attempted homicide, and all forms of assault (including aggravated and assault of an officer). This restriction meant that we excluded arson, sexual offences, and threats and intimidation. Separately, we also examined rates and odds of violent crime, by type of crime. Finally, we examined odds of violent crime specifically within 3 months, 6 months, and 12 months of the first diagnosis of depression to exclude patients who could have recovered from depression at the time of the outcome.

In relation to the sibling analyses, we excluded older siblings and younger siblings to avoid violence or depression in one group affecting the other, and those siblings with follow-up beginning before 2001 when outpatient data were unavailable.

We did cotwin control analyses to assess whether the observed association between depression and violent crime was due to common genetic or environmental influences.^43^ The twins were followed from the time of answering the questionnaire until any violent crime or end of study.^43^ First, standard Cox regression models were applied to estimate the association between sCESD score and violent crime, with adjustment for age (at the time of answering the questionnaire) and sex, with robust standard error accounting for twin clustering. Second, stratified Cox regression models were applied to estimate the association between sCESD score and violent crime in monozygotic and dizygotic twin pairs separately.

### Role of the funding source

The funders of the study had no role in the study design, data collection, data analysis, data interpretation, or writing of the report. SF had full access to all the data in the study and final responsibility for the decision to submit for publication.

## Results

We identified 47 158 individuals (17 249 men and 29 909 women) with outpatient diagnoses of depression between 2001 and 2009 (table 1). Men had a mean age at first diagnosis of 32 years (SD 10) and women of 31 years (10). The mean time of follow-up was 3·0 years (SD 2·3) for men and 3·2 years (2·3) for women.

**Table 1:** Descriptive data for risk factors in individuals with outpatient diagnoses of depression and control populations

	**Depression**		**Full siblings**		**Maternal half-siblings**		**Paternal half-siblings**		**General population controls**	
	Men (n=17 249)	Women (n=29 909)	Men (n=14 345)	Women (n=19 171)	Men (n=3519)	Women (n=3281)	Men (n=4517)	Women (n=4217)	Men (n=329 307)	Women (n=569 147)
**Sociodemographic factors**										
Age at 1st diagnosis, years	32 (10)	31 (10)	NA	NA	NA	NA	NA	NA	NA	NA
Income in lowest tertile	4835 (28%)	8093 (27%)	2298 (16%)	3422 (18%)	873 (25%)	895 (28%)	1267 (29%)	1229 (30%)	81 122 (25%)	143 941 (25%)
Born abroad	3520 (20%)	5417 (18%)	927 (7%)	1324 (7%)	47 (1%)	47 (1%)	138 (3%)	119 (3%)	60 530 (18%)	105 114 (18%)
Single	11 582 (67%)	17 862 (60%)	..	..	..	..	..	..	205 549 (60%)	321 175 (54%)
**Individual factors before diagnosis**										
Alcohol abuse	902 (5%)	836 (3%)	294 (2%)	198 (1%)	100 (3%)	48 (2%)	102 (2%)	55 (1%)	4818 (1%)	5563 (1%)
Drug abuse	715 (4%)	620 (2%)	173 (1%)	135 (1%)	72 (2%)	36 (1%)	74 (2%)	44 (1%)	2811 (1%)	2964 (1%)
Alcohol crime	565 (3%)	99 (<1%)	195 (1%)	32 (<1%)	96 (3%)	17 (<1%)	109 (2%)	10 (<1%)	2791 (<1%)	629 (<1%)
Drug crime	770 (5%)	209 (1%)	235 (2%)	73 (<1%)	97 (3%)	23 (1%)	109 (2%)	25 (1%)	3506 (1%)	1457 (<1%)
Alcohol or drug medication	253 (2%)	108 (<1%)	39 (<1%)	12 (<1%)	6 (<1%)	0	5 (<1%)	3 (<1%)	713 (<1%)	455 (<1%)
Any crime	6213 (36%)	3549 (12%)	3485 (24%)	1330 (7%)	1226 (35%)	336 (10%)	1514 (34%)	401 (10%)	67 942 (20%)	33 352 (6%)
Violent crime	2322 (14%)	572 (2%)	1080 (8%)	188 (1%)	408 (12%)	56 (2%)	501 (11%)	54 (1%)	19 068 (6%)	4071 (1%)
Non-violent crime	5749 (33%)	3282 (11%)	3268 (23%)	1262 (7%)	1152 (33%)	310 (9%)	1420 (31%)	384 (9%)	62 756 (18%)	31 485 (5%)
Self-harm	910 (5%)	2362 (8%)	278 (2%)	509 (3%)	94 (3%)	146 (5%)	101 (2%)	135 (3%)	5187 (2%)	11 114 (2%)

Data are mean (SD) or n (%). Income data were missing for 121 men and 253 women with depression, 134 men and 214 women with full siblings, 32 men and 23 women with maternal half-siblings, 72 men and 85 women with paternal half-siblings, and 14 341 men and 25 712 women in general population controls. NA=not applicable.

During follow-up, 641 men (3·7%) and 152 (0·5%) women with depression committed a violent crime, compared with 4097 (1·2%) men and 1059 (0·2%) women in age-matched and sex-matched controls.

Those individuals with depression were at 3-fold increased odds of violent crime compared with general population controls (table 2). The odds of violent crime were between 1·1 and 1·7 for both full and half-siblings compared with the general population controls. We noted a trend for higher odds ratios in the full siblings, as would be predicted by a genetic model. By comparing odds ratios in cases and sibling analyses, indirect comparisons can be made of cases compared with siblings (figure). These analyses showed that individuals with depression had two-fold increased odds of violent crime risk compared with their unaffected siblings (table 2).

**Table 2:** Adjusted odds ratios (aORs) and ratio of odds ratios (RORs) of violent crime in patients with depression, and in unaffected half-siblings and full siblings

		Patients with depression (n=47 158)*	**Paternal half-siblings (n=8734)**		**Maternal half-siblings (n=6800)**		**Full siblings (n=33 516)**	
			aOR (95% CI)	ROR (95% CI)	aOR (95% CI)	ROR (95% CI)	aOR (95% CI)	ROR (95% CI)
Overall		3·0 (2·8–3·3)	1·2 (1·1–1·4)	2·5 (2·2–2·9)	1·2 (1·1–1·4)	2·5 (2·2–2·8)	1·5 (1·3–1·6)	2·1 (1·8–2·4)
Sex								
	Male	3·1 (2·9–3·4)	1·2 (1·1–1·4)	2·5 (2·2–2·9)	1·2 (1·1–1·4)	2·5 (2·2–3·0)	1·4 (1·3–1·6)	2·2 (1·9–2·5)
	Female	2·8 (2·3–3·3)	1·2 (0·9–1·5)	2·4 (1·7–3·3)	1·2 (0·9–1·6)	2·3 (1·6–3·2)	1·7 (1·4–2·2)	1·6 (1·2–2·1)
Without previous								
	Alcohol or drugs	3·0 (2·8–3·3)	1·2 (1·1–1·4)	2·5 (2·2–2·9)	1·2 (1·0–1·3)	2·6 (2·2–3·0)	1·4 (1·3–1·6)	2·1 (1·9–2·4)
	Violent crime	3·0 (2·7–3·3)	1·2 (1·0–1·3)	2·6 (2·2–3·0)	1·2 (1·1–1·4)	2·4 (2·0–2·9)	1·5 (1·3–1·6)	2·1 (1·8–2·4)
	Any crime	2·7 (2·4–3·1)	1·1 (0·9–1·3)	2·4 (1·9–3·0)	1·3 (1·1–1·6)	2·0 (1·6–2·5)	1·4 (1·2–1·6)	1·9 (1·5–2·3)
	Self-harm	3·1 (2·8–3·4)	1·2 (1·1–1·3)	2·6 (2·2–3·0)	1·2 (1·1–1·4)	2·5 (2·2–2·9)	1·4 (1·3–1·6)	2·1 (1·9–2·4)
	All of above	2·6 (2·3–3·0)	1·1 (0·9–1·3)	2·3 (1·9–2·9)	1·3 (1·1–1·6)	2·0 (1·6–2·5)	1·4 (1·2–1·6)	1·9 (1·5–2·3)

All aORs (adjusted odds ratios) are compared with general population controls and matched by age and sex. aOR analyses are adjusted for low family income and being born abroad. ROR=ratio of odds ratios.

* Data are aOR (95% CI).

The odds of violent crime were similar for men with depression (compared with men without depression) and women with depression (compared with women without depression), and the relative risk increase remained significant when we excluded a history of previous violent and non-violent crimes, self-harm, and substance use disorders in both cases and controls (all p<0·0001; table 2). However, the presence of these pre-existing background factors led to notable changes in absolute risks of violent crime. A previous violent offending history had the largest effect—12·5% (n=291) of men and 3·8% (n=22) of women with this history committed a violent crime after a diagnosis of depression. The rates were further increased by addition of substance misuse and or self-harm (table 3). Combinations of risk factors increased absolute risk of violent crime to more than 15% in men, although the number of individuals with these combinations of risk factors was low (table 3).

**Table 3:** Prevalence of risk factors and rates of violent crime in individuals with depression with different background risk factors

	**Prevalence of risk factor by subgroup**		**Rate of violent crime by subgroup**	
	Men (n=17 249)	Women (n=29 909)	Men	Women
Overall	NA	NA	641 (3·7%)	152 (0·5%)
(1) Substance abuse	1466 (8·5%)	1353 (4·5%)	131 (8·9%)	28 (2·1%)
(2) Self-harm	910 (5·3%)	2362 (7·9%)	59 (6·5%)	34 (1·4%)
(3) Violent crime	2322 (13·5%)	572 (1·9%)	291 (12·5%)	22 (3·8%)
(1) and (2)	279 (1·6%)	423 (1·4%)	26 (9·3%)	9 (2·1%)
(1) and (3)	524 (3·0%)	115 (0·4%)	85 (16·2%)	6 (5·2%)
(2) and (3)	266 (1·5%)	127 (0·4%)	40 (15·0%)	10 (7·9%)
(1), (2), and (3)	123 (0·7%)	42 (0·1%)	20 (16·3%)	4 (9·5%)
Not (1), (2), or (3)	13 497 (78·2%)	26 245 (87·7%)	291 (2·2%)	89 (0·3%)

Data are n (%). NA=not applicable.

In those individuals with inpatient diagnoses between 2001 and 2009, the adjusted odds ratio of violent crime was higher at 5·7 (95% CI 5·2–6·2) than those with outpatient diagnoses only (3·0 [2·8–3·3]). In patients with depression, 3813 (8%) had a comorbid personality disorder; excluding those had no demonstrable effect on the adjusted odds ratio (aOR) of violence (aOR 3·1 [95% CI 2·8–3·3] compared with an aOR 3·0 [95% CI 2·8–3·3] in the whole sample). No significant differences were noted when outcomes were restricted to specific interpersonal crimes (homicide and attempted homicide, and all forms of assault; aOR 2·9, 95% CI 2·6–3·1). Rates and odds by type of violent crime are reported in the appendix p 1. Compared with 3·2 years of follow-up (793 violent crimes), non-significantly higher odds ratios were noted when we specifically examined violent crime within 3 months (aOR 3·6 [95% CI 2·8–4·5]; 87 violent crimes in depression sample), 6 months (3·3 [2·8–4·0]; 135 violent crimes), and 12 months (3·6 [3·2–4·1]; 346 violent crimes) of first diagnosis.

In sensitivity analyses of the sibling sample (appendix pp 2–4), we noted no significant differences after inclusion of only older siblings or only younger siblings, or exclusion of siblings with follow-up before 2001.

During a mean follow-up of 3·2 years, 575 (3·3%) men and 1287 (4·3%) women self-harmed (appendix p 5). These rates are comparable with the rates of violent crime described for men, but are higher for women. Self-harm in individuals with depression was increased compared with the general population (aOR 5·7, 95% CI 5·4–6·0). In the same period, 100 (0·6%) men and 41 (0·1%) women died by suicide (appendix p 5). Death by suicide was increased in patients with depression compared with the general population (aOR 6·7, 95% CI 5·5–8·1) and full siblings (ratio of odds ratio 2·9, 95% CI 2·2–3·8).

In the twin sample (9834 men and 13 186 women), the mean age at questionnaire completion was 32·7 years (SD 8·2) for men and 32·5 years (8·2) for women. The mean sCESD score was 6·9 (SD 5·1) for men and 7·6 (5·9) for women. The mean time of follow-up was 5·3 years (SD 0·5) for men and 5·4 years (0·3) for women. During follow-up, 73 men (0·7%) and 15 (0·1%) women committed a violent crime, with a mean time-to-event of 1·5 years (SD 1·0) for men and 1·6 years (1·0) for women.

The risk of violent crime significantly increased in those individuals with more depressive symptoms. In analyses of the association between depression and violent crime in co-twin analyses using standard Cox regression, the hazard ratio (HR; 95% CI) for all twins was 1·09 (1·06–1·13). For monozygotic twins, for which we used stratified Cox regression, the HR was 0·98 (0·82–1·18), and for dizygotic twins it was 1·07 (0·91–1·26). Statistical power was limited for further analyses of the twins by zygosity. In a sensitivity analysis of the twin sample, any crime was also significantly associated with increased depressive symptoms (HR 1·07, 95% CI 1·01–1·09).

## Discussion

In this population-based study, we investigated absolute and relative risks of violent crime after diagnosis of depression. In 47 158 individuals with depression, we noted three-fold increased odds of violent crime after adjustment for sociodemographic factors; this rate remained significantly elevated when we excluded patients with a previous history of substance abuse, any criminality, or self-harm. The association remained significant after adjustment for familial confounding, although the strength of the association was reduced. In other words, even after adjustment for genetic and early environmental factors, a diagnosis of depression modestly increased the risk of violent crime (panel).

Panel **Research in context**

**Systematic review**

We searched Medline from Jan 1, 1946, up to Nov 6, 2014, with no language restrictions, for articles on the association between depression and violence with genetically informed designs. The following search terms were used: (“depress*” or “unipolar*” or “affect*”) and (“sibling*” or “twin*”) and (“viol*” or “homicide” or “crim*”). We identified no studies. Further, we searched Medline specifically for systematic reviews (using the same search terms but without “sibling” or “twin”) and again identified no reviews.

**Interpretation**

To our knowledge, we have reported the first family-based studies of violence risk in depression. Our findings suggest that the odds of violent crime are elevated two to three fold after adjustment for familial, socioeconomic, and individual factors. Absolute rates of violent crime were low in the population-based investigation in men and substantially lower in women with depression, although certain subgroups with histories of substance misuse, violent crime, or both were associated with rates of more than 15% in men. Clinical guidelines should consider recommending violence risk assessment in certain subgroups with depression.

Comparisons of maternal and paternal half-siblings provide some indication whether the familial association is explained primarily by early environmental factors or whether it is genetic. If the risk attenuation had been stronger in the maternal half-siblings, this would suggest early environmental confounding because it is assumed that half-siblings share more environment if they share a mother rather than a father. In fact, the odds of violence were similar in maternal and paternal half-siblings, suggesting that the confounding is mostly genetic. The mediating mechanism translating genetic risk is unknown. One possibility is that comorbid personality disorders could partly explain the relationship. However, exclusion of those individuals with register-based diagnoses of personality disorder did not affect our results. Nevertheless, register diagnoses only capture a selection of individuals with personality problems and more sensitive markers of personality and disease could be more informative. Impulse control and affect regulation might be among the underlying mechanisms,^18^ which could explain the increased violence risk in other psychiatric disorders.

An important potential implication of our findings relates to interpretation of safety data for antidepressant medication. Anecdotally, antidepressants have been associated with self-harm and severe violence, which drew great attention in the public and media a decade ago.^44^ The reduction of antidepressant prescription to young people that followed failed to reduce rates of self-harm, with recent evidence suggesting that, in the USA, rates of self-harm actually increased.^45^ Although our study does not bear directly on the association between antidepressants and violence, it suggests that a diagnosis of depression will confound interpretation of the effects of treatment for depression on violence (and self-harm). Therefore pharmacovigilance data for antidepressants needs to be interpreted with great caution.

Our study benefits from several strengths. First, the use of population registers allowed us to obtain a large sample size. Furthermore, through register linkage we were able to exclude the more severe cases of depression, those with inpatient episodes. In a sensitivity analysis, we identified odds of violent crime that were around double in those with inpatient histories compared with outpatient groups, underscoring our decision to exclude these patients. In terms of generalisability, other work has showed that during 2006–08, 3·6% of depressed patients were admitted to hospital.^46^ Using genetically informed designs (half-siblings and siblings), we were able to examine the possibility of genetic and early environmental confounding behind the reported association between depression and violence. We used different methods to assess depression—clinical diagnoses in the population study and a continuous symptom measure in the twin investigation. Additionally, our sensitivity analyses showed similar patterns for violent crimes committed within 3 months, 6 months, and 12 months of diagnoses of depression, and separately for a related outcome—namely, all crimes.

Limitations of our study include restricted statistical power for our twin study to test for genetic confounding. Because we relied on patient registers, we did not have information about individuals with depression who present to primary care alone, and will not enter patient registers. Other Swedish research has showed that 47% of community individuals with Diagnostic and Statistical Manual-IV diagnoses of depression seek medical care. Of those who seek medical services, 26% receive general practitioner care alone, but these individuals have milder symptoms than those accessing psychiatric services.^47^ At the same time, focusing on those individuals who present to secondary care is advantageous because longer assessments and interventions can be offered than in primary care. To partly address this limitation, we additionally used twin data because this was a general (non-patient) sample. Another limitation is that we did not account for the effects of treatment because these data are only available from mid-2005, require different designs to examine,^48^ and will be investigated separately. A final limitation is that this study was done in one country, which could affect generalisability. However, the prevalence of violent assault in Sweden is similar to that in other high-income countries,^49^ and a Swedish study estimated the point prevalence of major depression at 5·2%,^50^ compared with 6·7% in the USA,^51^ and 6·3% in Australia.^52^

It is standard clinical practice that risk assessment in depression considers suicidal outcomes. Do the absolute risks of violence in depression presented here warrant changes to clinical guidelines, which currently make inconsistent recommendations regarding violent outcomes?^19^, ^20^, ^21^ The absolute risk of violent offending after diagnosis was 3·7% in the men with depression and 0·5% in women. Clinical depression guidelines from the USA state that violence and suicide risk should be monitored, and indicate that history of violence is a risk factor.^19^ By contrast, National Institutes for Health and Care Excellence (NICE) guidelines do not discuss risk of violence.^20^ The absolute rates of violence we have reported are certainly lower than those from similarly designed studies in other mental illnesses. However, we noted in some depressive subgroups (table 3), such as those with histories of both violent crime and substance abuse or self-harm, rates of violent crime of greater than 15% during the roughly 3 year follow-up. By comparison, absolute rates in men are higher in patients with schizophrenia at around 10% for similar time at risk^53^ and 8% in patients with with bipolar disorder.^54^ Guidelines are also inconsistent for these disorders. In schizophrenia, all newly diagnosed patients should be risk assessed according to NICE and US guidelines.^55^, ^56^ In bipolar disorder, this is not the recommendation.^38^

By contrast, clinical guidelines are consistent about the need for assessment of suicide risk in individuals with depression, schizophrenia, and bipolar disorder. The recent WHO publication on suicide prevention, for example, recommends detailed evaluations of suicide risk in depression.^22^ However, in our study, rates of self-harm for men were lower than rates of violent crime, and suicide mortality substantially lower also (appendix p 5). In terms of population effect, the association of violence with depression will be more important than with other diagnoses because depression is a more prevalent condition. Risk assessment remains prone to high false positive rates,^57^ and thus the consequences of classification of patients with depression into higher risk groups (by sex and pre-existing risk factors) should mitigate against potential harms. Indeed, those individuals identified as high risk could potentially benefit from psychological interventions as has been shown for suicidality in depression.^58^ Trials investigating whether drug or psychosocial interventions reduce violence could be of great interest in high risk samples.

In conclusion, we have shown an association between a diagnosis of depression and violent crime, which is independent of potential confounders and uses two complementary designs. The magnitude of the effects, certainly when compared with those for self-harm in the same population, suggest that assessment of the risk of violence should be considered routinely for some individuals with clinical depression and potentially included more consistently in clinical guidelines.

## References

[1] Chang C-K, Hayes RD, Perera G (2011). Life expectancy at birth for people with serious mental illness and other major disorders from a secondary mental health care case register in London. PLoS One.

[2] Cuijpers P, Vogelzangs N, Twisk J, Kleiboer A, Li J, Penninx BW (2014). Comprehensive meta-analysis of excess mortality in depression in the general community versus patients with specific illnesses. Am J Psychiatry.

[3] Hawton K, Houston K, Haw C, Townsend E, Harriss L (2003). Comorbidity of axis I and axis II disorders in patients who attempted suicide. Am J Psychiatry.

[4] Rosengren A, Hawken S, Ôunpuu S (2004). Association of psychosocial risk factors with risk of acute myocardial infarction in 11 119 cases and 13 648 controls from 52 countries (the INTERHEART study): case-control study. Lancet.

[5] Chapman DP, Perry GS, Strine TW (2005). The vital link between chronic disease and depressive disorders. Prev Chronic Dis.

[6] Moussavi S, Chatterji S, Verdes E, Tandon A, Patel V, Ustun B (2007). Depression, chronic diseases, and decrements in health: results from the World Health Surveys. Lancet.

[7] Oakley C, Hynes F, Clark T (2009). Mood disorders and violence: a new focus. Adv Psychiatr Treat.

[8] Eliason S (2009). Murder-suicide: a review of the recent literature. J Am Acad Psychiatry Law.

[9] Coid J, Yang M, Roberts A (2006). Violence and psychiatric morbidity in a national household population- a report from the British Household Survey. Am J Epidemiol.

[10] Wallace C, Mullen P, Burgess P, Palmer S, Ruschena D, Browne C (1998). Serious criminal offending and mental disorder. Br J Psychiatry.

[11] Monahan J, Steadman H, Tonry M, Morris N (1983). Crime and Justice: an annual review of research.

[12] Arseneault L, Moffitt TE, Caspi A, Taylor PJ, Silva PA (2000). Mental disorders and violence in a total birth cohort: results from the Dunedin Study. Arch Gen Psychiatry.

[13] Elbogen EB, Johnson SC (2009). The intricate link between violence and mental disorder: results from the National Epidemiologic Survey on Alcohol and Related Conditions. Arch Gen Psychiatry.

[14] Van Dorn R, Volavka J, Johnson N (2012). Mental disorder and violence: is there a relationship beyond substance use?. Soc Psychiatry Psychiatr Epidemiol.

[15] Ten Have M, de Graaf R, van Weeghel J, van Dorsselaer S (2014). The association between common mental disorders and violence: to what extent is it influenced by prior victimization, negative life events and low levels of social support?. Psychol Med.

[16] Brennan PA, Mednick SA, Hodgins S (2000). Major mental disorders and criminal violence in a Danish birth cohort. Arch Gen Psychiatry.

[17] Witt K, Van Dorn R, Fazel S (2013). Risk factors for violence in psychosis: systematic review and meta-regression analysis of 110 Studies. PLoS One.

[18] Nestor PG (2002). Mental disorder and violence: personality dimensions and clinical features. Am J Psychiatry.

[19] American Psychiatric Association (APA) (2010). Practice guideline for the treatment of patients with major depressive disorder.

[20] National Institute for Health and Care Excellence Depression in adults: the treatment and management of depression in adults [CG90]. https://www.nice.org.uk/guidance/cg90/resources/guidance-depression-in-adults-pdf.

[21] Ellis P, Royal Australian and New Zealand College of Psychiatrists Clinical Practice Guidelines Team for Depression (2004). Australian and New Zealand clinical practice guidelines for the treatment of depression. Aust N Z J Psychiatry.

[22] WHO (2014). Preventing suicide: a global imperative.

[23] Statistics Sweden (2005). Flergenerationsregistret 2004: En beskrivning av innehåll och kvalitet. [The Multi-Generation Register 2004: a description of content and quality].

[24] Lichtenstein P, Sullivan PF, Cnattingius S (2006). The Swedish Twin Registry in the third millennium: an update. Twin Res Hum Genet.

[25] Lichtenstein P, Tuvblad C, Larsson H, Carlström E (2007). The Swedish twin study of child and adolescent development: the TCHAD-study. Twin Res Hum Genet.

[26] Swedish National Board of Health and Welfare Patientregistret. http://www.socialstyrelsen.se/register/halsodataregister/patientregistret.

[27] Carpenter JS, Andrykowski MA, Wilson J (1998). Psychometrics for two short forms of the Center for Epidemiologic Studies-Depression Scale. Issues Ment Health Nurs.

[28] Sellgren C, Landén M, Lichtenstein P, Hultman C, Långström N (2011). Validity of bipolar disorder hospital discharge diagnoses: file review and multiple register linkage in Sweden. Acta Psychiatr Scand.

[29] Ekholm B, Ekholm A, Adolfsson R (2005). Evaluation of diagnostic procedures in Swedish patients with schizophrenia and related psychoses. Nordic J Psychiatry.

[30] Ludvigsson J, Andersson E, Ekbom A (2011). External review and validation of the Swedish national inpatient register. BMC Public Health.

[31] Bergman B, Belfrage H, Grann M (1999). Mentally disordered offenders in Sweden: forensic and general psychiatric diagnoses. Am J Forensic Psychiatry.

[32] Fazel S, Grann M (2006). The population impact of severe mental illness on violent crime. Am J Psychiatry.

[33] Becker HK, Hjellemo EO (1976). Justice in modern Sweden.

[34] National Board of Health and Welfare Cause of Death Register. Stockholm. http://www.socialstyrelsen.se/register/dodsorsaksregistret.

[35] Neeleman J, Wessely S (1997). Changes in classification of suicide in England and Wales: time trends and associations with coroners' professional backgrounds. Psychol Med.

[36] Fazel S, Långström N, Hjern A, Grann M, Lichtenstein P (2009). Schizophrenia, substance abuse, and violent crime. JAMA.

[37] Fazel S, Lichtenstein P, Grann M, Långström N (2011). Risk of violent crime in individuals with epilepsy and traumatic brain injury: a 35-Year Swedish Population Study. PLoS Med.

[38] Fazel S, Lichtenstein P, Grann M, Goodwin G, Långström N (2010). Bipolar disorder and violent crime: new evidence from population-based longitudinal studies and systematic review. Arch Gen Psychiatry.

[39] Klein-Geltink J, Rochon P, Dyer S, Laxer M, Anderson G (2007). Readers should systematically assess methods used to identify, measure and analyze confounding in observational cohort studies. J Clin Epidemiol.

[40] Altman DG, Bland JM (2003). Interaction revisited: the difference between two estimates. BMJ.

[41] Newton-Howes G, Tyrer P, Johnson T (2006). Personality disorder and the outcome of depression: meta-analysis of published studies. Br J Psychiatry.

[42] Yu R, Geddes JR, Fazel S (2012). Personality disorders, violence, and antisocial behavior: a systematic review and meta-regression analysis. J Pers Disord.

[43] Kendler KS, Neale MC, MacLean CJ, Heath AC, Eaves LJ, Kessler RC (1993). Smoking and major depression: a causal analysis. Arch Gen Psychiatry.

[44] Healy D, Herxheimer A, Menkes DB (2006). Antidepressants and violence: problems at the interface of medicine and law. PLoS Med.

[45] Lu CY, Zhang F, Lakoma MD (2014). Changes in antidepressant use by young people and suicidal behavior after FDA warnings and media coverage: quasi-experimental study. BMJ.

[46] Ekman M, Granström O, Omerov S, Jacob J, Landen M (2013). The societal cost of depression: evidence from 10,000 Swedish patients in psychiatric care. J Affect Disord.

[47] Wallerblad A, Möller J, Forsell Y (2012). Care-seeking pattern among persons with depression and anxiety: a population-based study in Sweden. Int J Family Med.

[48] Fazel S, Zetterqvist J, Larsson H, Långström N, Lichtenstein P (2014). Antipsychotics, mood stabilisers, and risk of violent crime. Lancet.

[49] Heiskanen M, Harrendorf S, Heiskanen M, Malby S (2010). International statistics on crime and justice.

[50] Johansson R, Carlbring P, Heedman Å, Paxling B, Andersson G (2013). Depression, anxiety and their comorbidity in the Swedish general population: point prevalence and the effect on health-related quality of life. PeerJ.

[51] Kessler RC, Chiu WT, Demler O, Walters EE (2005). Prevalence, severity, and comorbidity of 12-month DSM-IV disorders in the National Comorbidity Survey Replication. Arch Gen Psychiatry.

[52] Andrews G, Henderson S, Hall W (2001). Prevalence, comorbidity, disability and service utilisation. Overview of the Australian National Mental Health Survey. Br J Psychiatry.

[53] Fazel S, Wolf A, Palm C, Lichtenstein P (2014). Violent crime, suicide, and premature mortality in patients with schizophrenia and related disorders: a 38-year total population study in Sweden. Lancet Psychiatry.

[54] Webb R, Lichtenstein P, Larsson H, Geddes J, Fazel S (2014). Suicide, hospital-presenting suicide attempts, and criminality in bipolar disorder: examination of risk for multiple adverse outcomes. J Clin Psychiatry.

[55] National Institute for Health and Care Excellence (2014). Psychosis and schizophrenia in adults: treatment and management [CG178]. https://www.nice.org.uk/guidance/cg178/resources/guidance-psychosis-and-schizophrenia-in-adults-treatment-and-management-pdf.

[56] American Psychiatric Association (APA) (2004). Practice guideline for the treatment of patients with schizophrenia.

[57] Fazel S, Singh JP, Doll H, Grann M (2012). Use of risk assessment instruments to predict violence and antisocial behaviour in 73 samples involving 24 827 people: systematic review and meta-analysis. BMJ.

[58] Tarrier N, Taylor K, Gooding P (2008). Cognitive-behavioral interventions to reduce suicide behavior: a systematic review and meta-analysis. Behav Modif.

