# Supplementary material for: Depression and violence: a Swedish population study
Source: Lancet Psychiatry. 2015 Oct;2(3):224–32. doi: 10.1016/S2215-0366(14)00128-X (PMC4520382; doi:10.1016/S2215-0366(14)00128-X)
Supplement: Supplementary appendix [file mmc1.pdf]

THE LANCET Psychiatry

Supplementary appendix

This appendix formed part of the original submission and has been peer reviewed. We post it as supplied by the authors.

Supplement to: Fazel S, Wolf A, Chang Z, Larsson H, Goodwin GM, Lichtenstein P. Depression and violence: a Swedish population study. *Lancet Psychiatry* 2015; **2:** 244–32.

Appendix Table 1. First crime after diagnosis.

Type of violent crime	MEN (n=17,249)			WOMEN (n=29,909)		
	n (% of those with depression)	% of violent offenders	aOR (95% CI)	n (% of those with depression)	% of violent offenders	aOR (95% CI)
**Homicide**	5 (0.03%)	0.8%	3.0 (1.1-7.8)	0 (0%)	0%	- (---)
**Assault**	426 (2.5%)	66.5%	2.9 (2.6-3.2)	114 (0.4%)	75.0%	2.7 (2.2-3.3)
**Sexual offences**	21 (0.1%)	3.3%	1.8 (1.1-2.7)	2 (0.01%)	1.3%	5.2 (1.0-25.8)
**Robbery**	31 (0.2%)	4.8%	3.0 (2.0-4.4)	4 (0.01%)	2.6%	2.8 (1.0-8.3)
**Arson**	2 (0.01%	0.3%	2.0 (0.5-8.8)	1 (0.01%)	0.7%	1.2 (0.2-10.0)
**Threats & harassment**	231 (1.3%)	36.0%	4.1 (3.5-4.7)	43 (0.1%)	28.3%	3.3 (2.4-4.5)

Note: All aORs (adjusted odds ratios) are compared with general population controls and matched by age and sex. aOR analyses are adjusted for low family income and being born abroad. Homicide includes attempted homicide. Assault includes aggravated assault, common assault, and assaulting an officer. Some cases committed more than one type of crime within a particular incident.

Appendix Table 2a: Adjusted odds ratios of violent crime in patients with depression and unaffected siblings compared with general population controls, using 2001-2009 follow-up only.

2001-2009 follow-up	Paternal half-siblings n=2,477		Maternal half-siblings n=2,070		Full siblings n=23,372	
	**aOR**	**ROR**	**aOR**	**ROR**	**aOR**	**ROR**
**Overall**	1.3 (1.0-1.7)	2.3 (1.8-3.0)	1.4 (1.1-1.8)	2.2 (1.7-2.9)	1.4 (1.3-1.6)	2.1 (1.8-2.5)
**Sex**						
**Male**	1.3 (1.0-1.7)	2.4 (1.8-3.2)	1.4 (1.1-1.9)	2.2 (1.6-2.9)	1.4 (1.2-1.6)	2.2 (1.9-2.6)
**Female**	1.3 (0.6-2.6)	2.1 (1.1-4.3)	1.2 (0.6-2.2)	2.4 (1.2-4.6)	1.6 (1.2-2.3)	1.7 (1.2-2.5)
**Without previous:**						
**Alc/drug**	1.3 (1.0-1.8)	2.3 (1.7-3.1)	1.2 (0.9-1.7)	2.5 (1.8-3.3)	1.4 (1.2-1.6)	2.2 (1.8-2.5)
**Violent crime**	1.3 (0.9-1.8)	2.4 (1.7-3.4)	1.4 (1-1.9.0)	2.2 (1.6-3.1)	1.4 (1.2-1.7)	2.1 (1.7-2.5)
**Any crime**	0.9 (0.5-1.5)	3.0 (1.8-5.0)	1.3 (0.8-2.0)	2.1 (1.3-3.3)	1.5 (1.2-1.8)	1.8 (1.4-2.3)
**Self-harm**	1.3 (1.0-1.7)	2.4 (1.8-3.1)	1.4 (1.0-1.8)	2.3 (1.7-3.0)	1.4 (1.2-1.6)	2.2 (1.9-2.6)
**All of above**	1.0 (0.6-1.6)	2.7 (1.6-4.5)	1.4 (0.9-2.2)	1.9 (1.2-3.0)	1.4 (1.2-1.7)	1.9 (1.4-2.4)

All aORs (adjusted odds ratios) are compared with general population controls and matched by age and sex. aOR analyses are adjusted for low family income and being born abroad. ROR=ratio of odds ratios.

Appendix Table 2b: Adjusted odds ratios of violent crime in patients with depression and unaffected siblings compared with general population controls, using younger siblings only

Younger siblings only	Paternal half-siblings n=975		Maternal half-siblings n=808		Full siblings n=7,691	
	**aOR**	**ROR**	**aOR**	**ROR**	**aOR**	**ROR**
**Overall**	1.1 (0.5-2.2)	2.8 (1.4-5.9)	1.2 (0.6-2.2)	2.6 (1.4-5.1)	1.2 (0.9-1.6)	2.5 (1.9-3.5)
**Gender**						
**Male**	1.3 (0.6-2.7)	2.4 (1.1-5.0)	1.2 (0.6-2.4)	2.6 (1.3-5.2)	1.4 (1.0-1.8)	2.3 (1.7-3.2)
**Female**	- (---)	- (---)	0.9 (0.1-6.8)	3.2 (0.4-24.8)	0.2 (0.0-1.4)	13.8 (1.9-100)
**Without previous:**						
**Alc/drug**	1.1 (0.5-2.6)	2.7 (1.2-6.2)	0.9 (0.4-2.0)	3.4 (1.5-7.9)	1.3 (0.9-1.7)	2.4 (1.7-3.3)
**Violent crime**	1.2 (0.5-3.1)	2.4 (1.0-6.1)	1.2 (0.5-2.9)	2.6 (1.0-6.5)	1.1 (0.7-1.6)	2.8 (1.8-4.2)
**Any crime**	0.8 (0.2-3.2)	3.5 (0.8-14.6)	1.1 (0.3-3.6)	2.5 (0.7-8.2)	1.0 (0.6-1.7)	2.6 (1.5-4.3)
**Self-harm**	1.1 (0.5-2.3)	2.8 (1.3-6.2)	1.0 (0.5-2.1)	3.0 (1.4-6.3)	1.2 (0.9-1.6)	2.6 (1.9-3.6)
**All of above**	1.0 (0.2-4.2)	2.6 (0.6-11.2)	1.1 (0.3-3.8)	2.3 (0.7-7.8)	0.9 (0.5-1.6)	2.9 (1.6-5.1)

All aORs (adjusted odds ratios) are compared with general population controls and matched by age and sex. aOR analyses are adjusted for low family income and being born abroad. ROR=ratio of odds ratios.

Appendix Table 2c: Adjusted odds ratios of violent crime in patients with depression and unaffected siblings compared with general population controls, using older siblings only

Older siblings only	Paternal half-siblings n=7,759		Maternal half-siblings n=5,992		Full siblings n=25,825	
	**OR**	**ROR**	**OR**	**ROR**	**OR**	**ROR**
**Overall**	1.2 (1.1-1.4)	2.5 (2.2-2.8)	1.2 (1.1-1.4)	2.5 (2.2-2.8)	1.5 (1.4-1.6)	2.0 (1.8-2.3)
**Gender**						
**Male**	1.2 (1.1-1.4)	2.6 (2.2-2.9)	1.2 (1.1-1.4)	2.5 (2.2-3.0)	1.4 (1.3-1.6)	2.2 (1.9-2.5)
**Female**	1.2 (0.9-1.6)	2.3 (1.7-3.2)	1.2 (0.9-1.7)	2.2 (1.6-3.2)	1.9 (1.5-2.4)	1.4 (1.1-1.9)
**Without previous:**						
**Alc/drug**	1.2 (1.1-1.4)	2.5 (2.2-2.9)	1.2 (1.1-1.4)	2.5 (2.2-2.9)	1.4 (1.3-1.6)	2.1 (1.8-2.4)
**Violent crime**	1.2 (1.0-1.3)	2.6 (2.2-3.0)	1.2 (1.1-1.4)	2.4 (2.0-2.9)	1.5 (1.3-1.7)	2.0 (1.7-2.3)
**Any crime**	1.1 (0.9-1.3)	2.4 (1.9-3.0)	1.3 (1.1-1.6)	2.0 (1.6-2.5)	1.5 (1.3-1.7)	1.8 (1.5-2.2)
**Self-harm**	1.2 (1.1-1.3)	2.6 (2.2-3.0)	1.2 (1.1-1.4)	2.5 (2.2-2.9)	1.5 (1.3-1.6)	2.1 (1.8-2.4)
**All of above**	1.1 (0.9-1.3)	2.3 (1.9-2.9)	1.3 (1.1-1.6)	2.0 (1.6-2.5)	1.4 (1.2-1.7)	1.8 (1.5-2.2)

All aORs (adjusted odds ratios) are compared with general population controls and matched by age and sex. aOR analyses are adjusted for low family income and being born abroad. ROR=ratio of odds ratios.

Appendix Figure 1. Absolute rates of self-harm, violent crime, and suicide in patients with depression, by sex.

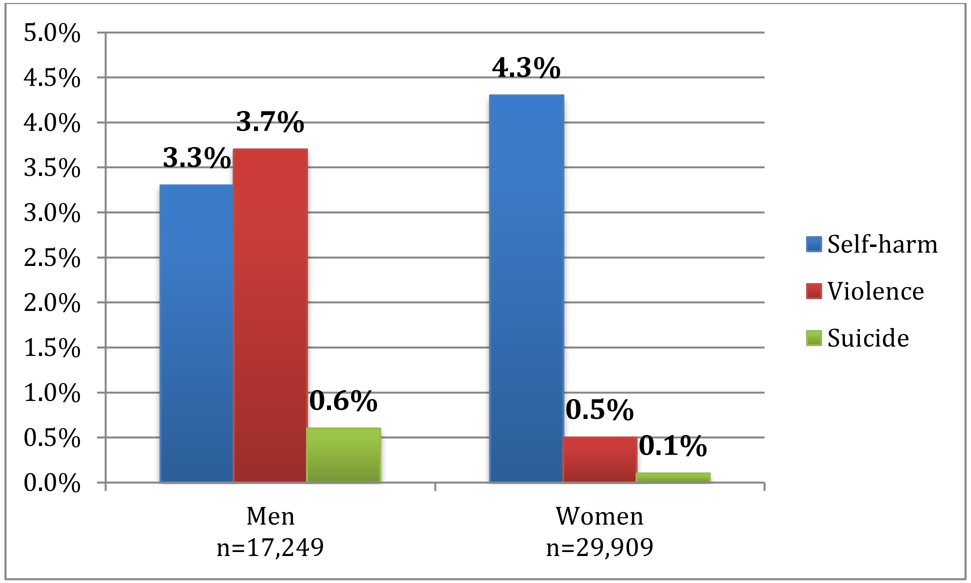

Note: Mean follow-up of 3.0 years (SD 2.3) for men, 3.2 years (2.3) for women.
